# Social Media, Endometriosis, and Evidence-Based Information: An Analysis of Instagram Content

**DOI:** 10.3390/healthcare12010121

**Published:** 2024-01-04

**Authors:** Hannah Adler, Monique Lewis, Cecilia Hoi Man Ng, Cristy Brooks, Mathew Leonardi, Antonina Mikocka-Walus, Deborah Bush, Alex Semprini, Jessica Wilkinson-Tomey, George Condous, Nikhil Patravali, Jason Abbott, Mike Armour

**Affiliations:** 1 Centre for Social and Cultural Research, Griffith University, Gold Coast, QLD 4215, Australia; monique.lewis@griffith.edu.au; 2 Division of Obstetrics and Gynaecology, School of Clinical Medicine, Faculty of Medicine and Health, University of New South Wales, Sydney, NSW 2052, Australia; cecilia.ng@unsw.edu.au (C.H.M.N.); j.abbott@unsw.edu.au (J.A.); 3 Gynaecological Research and Clinical Evaluation (GRACE) Unit, Royal Hospital for Women, Sydney, NSW 2031, Australia; 4 National Endometriosis Clinical and Scientific Trials (NECST) Network, University of New South Wales, Sydney, NSW 2052, Australia; 5 School of Health Sciences, Western Sydney University, Sydney, NSW 2751, Australia; 6 Translational Health Research Institute, Western Sydney University, Sydney, NSW 2751, Australia; m.armour@westernsydney.edu.au; 7 Department of Obstetrics and Gynecology, McMaster University, Hamilton, ON L8S 4LB, Canada; 8 Robinson Research Institute, University of Adelaide, Adelaide, SA 5006, Australia; 9 School of Psychology, Deakin University, Melbourne, VIC 3125, Australia; 10 World Endometriosis Organisations (WEO), Christchurch 8013, New Zealand; 11 Medical Research Institute of New Zealand, Wellington 6021, New Zealand; 12 School of Biological Sciences, Victoria University of Wellington, Wellington 6021, New Zealand; 13 NICM Health Research Institute, Western Sydney University, Sydney, NSW 2145, Australia; 14 Endometriosis Ultrasound and Advanced Endosurgery Unit, Sydney Medical School Nepean, Sydney, NSW 2747, Australia; 15 Nepean Hospital, University of Sydney, Sydney, NSW 2747, Australia; 16 School of Medicine, University of Sydney, Sydney, NSW 2747, Australia; 17 Monash IVF, Sydney, NSW 2747, Australia; 18 Mildura Private Hospital, Mildura, VIC 3500, Australia

**Keywords:** endometriosis, social media, Instagram, pelvic pain, advocacy

## Abstract

Social media platforms are used for support and as resources by people from the endometriosis community who are seeking advice about diagnosis, education, and disease management. However, little is known about the scientific accuracy of information circulated on Instagram about the disease. To fill this gap, this study analysed the evidence-based nature of content on Instagram about endometriosis. A total of 515 Instagram posts published between February 2022 and April 2022 were gathered and analysed using a content analysis method, resulting in sixteen main content categories, including “educational”, which comprised eleven subcategories. Claims within educational posts were further analysed for their evidence-based accuracy, guided by a process which included fact-checking all claims against the current scientific evidence and research. Of the eleven educational subcategories, only four categories (cure, scientific article, symptoms, and fertility) comprised claims that were at least 50% or greater evidence-based. More commonly, claims comprised varying degrees of evidence-based, mixed, and non-evidence-based information, and some categories, such as surgery, were dominated by non-evidence-based information about the disease. This is concerning as social media can impact real-life decision-making and management for individuals with endometriosis. Therefore, this study suggests that health communicators, clinicians, scientists, educators, and community groups trying to engage with the endometriosis online community need to be aware of social media discourses about endometriosis, while also ensuring that accurate and translatable information is provided.

## 1. Introduction

Endometriosis is a common but under-recognised chronic disease. Globally, it has been estimated to affect approximately one in ten women, girls, and transgender and gender-diverse people [1]. In Australia, recent estimates suggest that by the age of 44, one in nine Australian women, girls, and transgender and gender-diverse people are diagnosed with endometriosis [2]. It is often associated with significant dysmenorrhea, non-cyclical pelvic pain, dyspareunia, subfertility, and fatigue [3]. Diagnostic delay ranges between 6.4 [4] and 8 years [3], due to limited knowledge in the general public and health sector; the normalisation and diversity of symptoms; and limitations to non-invasive biomarkers and imaging techniques [5,6]. Once diagnosed, there are several options for management including surgery and medical therapies, but satisfaction with current treatments is low, with less than 25% of those with endometriosis in Australia feeling satisfied with their symptom management [7].

In 2018, as a result of patient-advocacy and collaboration with Australian clinicians, researchers, and policy makers, the National Action Plan for Endometriosis (NAPE) was established. Similar action plans in the U.K. [8,9], Canada [10], and France [11] are being developed, following in Australia’s footsteps. A key outcome from the Australian NAPE was the production and release of the *Australian clinical practice guideline for the diagnosis and management of endometriosis* by the Royal Australian and New Zealand College of Obstetricians and Gynaecologists (RANZCOG). The aim of the guideline was to provide the best available scientific evidence to assist health professionals with the detection, diagnosis, treatment, and management of endometriosis and the related condition, adenomyosis, and to provide the best possible quality of care [12]. Incidentally, the release of the Australian clinical practice guidelines coincided with the release of the updated European Society of Human Reproduction and Embryology (ESHRE) endometriosis guidelines [13] the following year. However, following the release of these guidelines, people with endometriosis and advocates took to social media to voice their concerns and criticisms of the new recommendations, including concerns related to symptoms, referral to care, detection of endometriosis via imaging scans, surgical management, and treatment, through the hashtag #changetheguidelines. Such advocacy on social media is a common feature of the endometriosis community [14] as seen through other events such as the annual Endometriosis Awareness Month campaign, which is aimed at increasing awareness of endometriosis [15].

However, the use of social media by the endometriosis community moves beyond the purpose of activism and advocacy. Rather, these platforms are also used for support and as resources, providing a place of community for people who are seeking advice about diagnosis, education, and disease management [6,16,17,18,19,20]. Survey and interview data have shown the important role social media plays in the process of diagnosis and learning about endometriosis [18], and the influence these platforms have on real-life decision-making and management of the disease [20], highlighting the importance of accurate information. For example, accurate information on the symptoms and presentation of the disease, and how an accurate diagnosis may be obtained, may assist with reducing the delay between symptom onset and seeking medical help. Yet, existing research has found that the evidence-based nature of content on these platforms varies, and while content about endometriosis on Facebook is largely evidence-based [19], incorrect and biased information on social media is a concern for those from the endometriosis community [20]. For instance, content on Instagram often contains general or vague knowledge about endometriosis [15], and inaccurate information about the disease is commonly found online [16,21]. Therefore, while research to date has investigated the evidence-based nature of content on Facebook about endometriosis, and described the general nature of Instagram content, little is known about the scientific accuracy of information circulated on Instagram. This is concerning given the pivotal role that Instagram in particular may play for those with endometriosis [6,15,17,19]. Thus, this study aimed to fill the evidence gap by determining what proportion of user-generated content on Instagram was evidence-based, and explored reasons for any discrepancies between peer-reviewed evidence and statements of fact within posts.

## 2. Materials and Methods

Content analysis was chosen as the method for this study, as it is a systematic and replicable approach to data categorisation [22] that is frequently used in health and wellness studies to analyse content on Instagram [23,24,25,26,27,28]. While Australian-based studies have investigated the type of content posted on Instagram about endometriosis [15], to the best of the authors’ knowledge, no studies have analysed the evidence-based nature of this content. The design of this project was based on the approach taken by Towne and colleagues [19], which involved using content analysis to organise the social media posts into content categories, followed by a secondary analysis focused on determining the scientific accuracy of the content. This study applied a similar approach to analysing Instagram posts.

### 2.1. Data Collection

The top posts feature of Instagram [26] was used to identify the most popular content associated with #changetheguidelines, which enabled the gathering of content with the broadest public reach. The hashtag of #changetheguidelines was chosen as the preliminary hashtag for analysis, as it was the most central to this research. A new Instagram account was created for this study, to ensure that history or pre-existing usage did not affect the data. Instagram uses multiple algorithms, and the platform gathers data on its users based on the variety of ways they engage with different features (e.g., via views, posts, hashtags, shares, and comments). By creating a new Instagram account, it was ensured that, as much as possible, top posts associated with hashtags were based on the popularity of the post, including the amount of likes, comments, and shares [29].

Over the course of a week, two coders used the top posts feature, searching #changetheguidelines to explore posts made between February and April 2022 (to include the month before, during, and after Endometriosis Awareness Month). Drawing on existing methods used in similar research [23], the two coders then listed all relevant hashtags associated with the posts, and the two most prominent endometriosis-related hashtags, #endowarrior and #endometriosisawarenessmonth, were also chosen as data collection points.

The two coders then searched each hashtag, and again using the top posts feature, downloaded all posts made between February and April 2022. Duplicates and videos were not included in the final sample. A total of 515 posts were collected and de-identified from #changetheguidelines (n = 65), #endowarrior (n = 198), and #endometriosisawarenessmonth (n = 252) for analysis. The unit of analysis for each Instagram post included the visual image used, the caption, and the hashtag(s) [25]. Posts were de-identified by placing the content of the post within a spreadsheet, with identifiable information such as account usernames removed.

### 2.2. Analysis

#### 2.2.1. Content Categories

The first stage of analysis included creating a preliminary abductive codebook of content categories, with prior deductive content categories being adapted from Towne and colleagues’ study on Facebook content about endometriosis [19]. However, some of these prior categories needed to be modified to ensure relevance to this research. For instance, categories such as “recipes” were not relevant to the research context, and epidemiology and pathophysiology were separated into two distinct categories. Once the deductive content categories had been identified, a pilot study was completed. For the pilot, five coders, including two lead researchers, coded 10 posts from each hashtag (n = 30 posts in total), to develop the inductive content categories. These content categories were then finalised and consolidated with the a priori content categories to form the codebook. Two coders then worked together to code the remaining posts and discuss newly emerging categories, ensuring consistency in the results and measuring the level of agreements through reflexivity, discussion, and sticking close to the codebook and data, as used elsewhere [30]. 

This resulted in 16 different content categories: education/information-sharing, advocacy, non-endometriosis specific, resources, emotional support, medical distrust, discussion, affirmation, promotional, diet and lifestyle, pregnancy, humour, events, economics, other, and surveys. Following the design of Towne and colleagues [19], posts were considered educational in nature if they included facts about endometriosis to convey information or to educate the reader. Posts coded as educational/information-sharing were the focus of this study and underwent a secondary analysis, which focused on determining the evidence-based accuracy of the claims being made. 

#### 2.2.2. Post Accuracy

Firstly, two coders isolated and identified all posts within the education and information-sharing categories, and isolated claims about endometriosis from within the posts. These claims were then organised into their relevant content category for evidence-based fact checking. After this process was complete, two coders with relevant specialisations were chosen for each category (e.g., surgical claims were assigned to those with clinical and academic expertise in the area of endometriosis surgery). This stage of analysis included 14 coders, with specialisations ranging from gynaecological surgeons and medical doctors to researchers (including scientists) in endometriosis and women’s and reproductive health, health psychology, and health communication. Each claim was read by these two independent coders to determine the post’s accuracy [19]. To ensure reliability, each coder pair met to compare and finalise their results. If the two coders could not agree on the evidence-based nature of a certain claim, a third coder was consulted who considered the evidence from both coders, while completing their own research relevant to the claim. 

Posts were assessed for accuracy by searching through peer-reviewed academic literature. Both Google Scholar and Medline were used. Google Scholar was used as it was more likely to pick up journal articles which were not indexed in Medline, thereby capturing the broadest range of potential “evidence”. Secondary searches within Medline using PubMed were undertaken if authors were concerned about potentially missing articles. Evidence was considered according to the Levels of Evidence for Therapeutic Studies, with systematic reviews/meta-analysis providing the “best” evidence [31]. Claims were assessed as follows: “evidence-based” if it was clear that this was supported by peer-reviewed journal articles (including original research), meta-analyses, and systematic reviews; “non-evidenced-based” in which there was no support found at all for the claim; or “mixed” in cases in which the wording or phraseology used meant that part of the claim was evidence-based and the other part of the claim was ambiguous, contradictory, or incorrect.

## 3. Results

### 3.1. Content Categories

**Figure 1:**
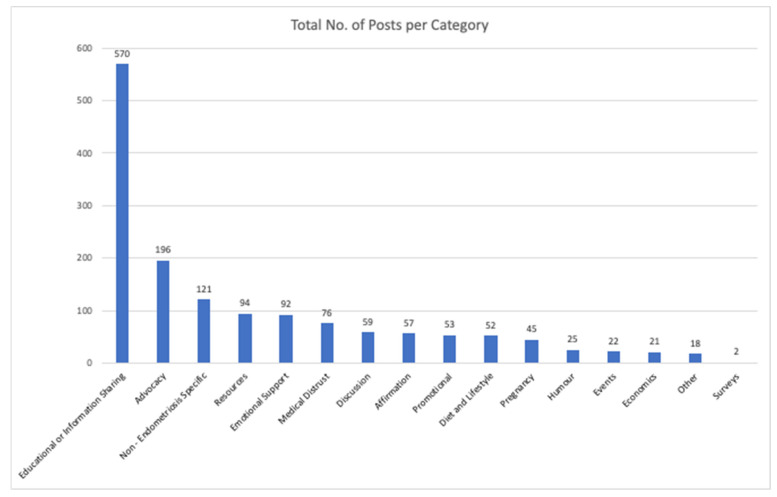
Total number of posts per content category.

In total, 515 posts were analysed and categorised into their relevant content categories. Figure 1 presents these findings, demonstrating the different content categories and the total number of posts that comprised each category. The category which included the greatest number of posts was education/information-sharing (when not broken into its subcategories), as posts included information that related to multiple aspects of endometriosis (n = 570). Other content categories included information about advocacy (n = 196), resources (n = 94), emotional support (n = 92), and medical distrust (n = 76).

**Figure 2:**
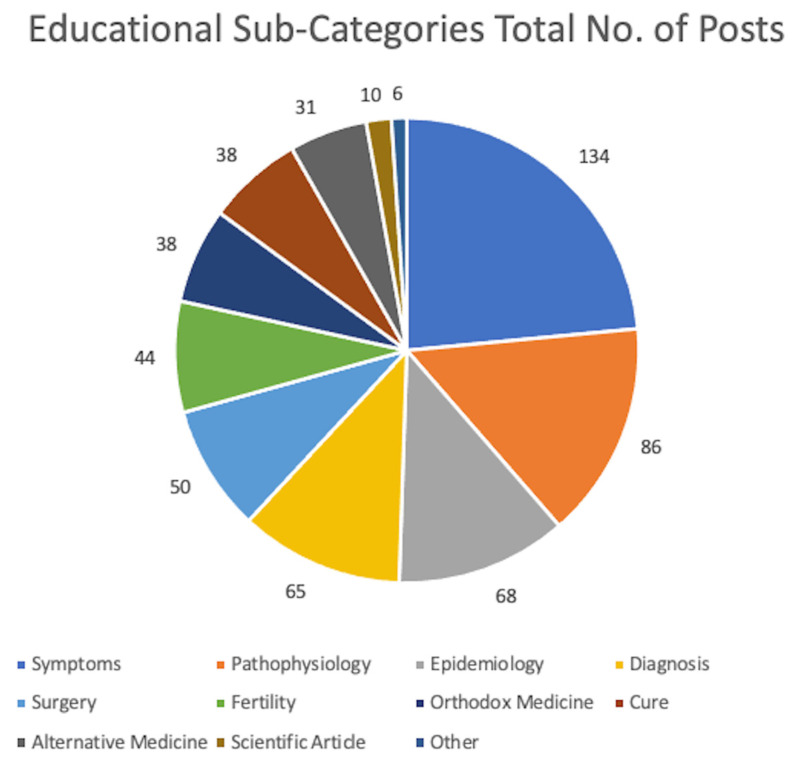
Total number of posts per Educational and Information-Sharing content category.

The breakdown of the education or information-sharing category into sub-categories (Figure 2) indicates that the highest number of posts referred to symptoms (n = 134), followed by pathophysiology (n = 86) and epidemiology (n = 68). 

Diagnosis also followed closely (n = 65), with many posts addressing the diagnostic process and the number of people diagnosed with endometriosis each year. There was not a substantial difference between the frequency of posts about orthodox medicine (n = 38) or complementary and alternative medicines (n = 31) regarding medical and pharmaceutical treatment for endometriosis. Claims to fertility (n = 44) and surgery (n = 50) were also at similar frequencies, with fewer claims regarding cure (n = 38) and scientific articles (n = 10).

### 3.2. Evidence-Based Nature of Posts

The secondary stage of analysis assessed the extent of the evidence-based nature of the Instagram posts within the educational content categories (Table 1); particularly, analysis of the individual claims made within each of these posts was performed. This included listing all claims made per educational category and then assessing each claim as “evidence-based”, “non-evidence-based”, or “mixed evidence”. The total number of claims under each category are listed in Table 1 below, and an example of the evidence-based nature of the posts can be seen in Table 2. 

The examples of claims provided in Table 2 demonstrate some of the differences in language between evidence-based, non-evidence-based, and mixed information. While a more comprehensive analysis of the framings and latent meanings within the claims, beyond their evidence-based nature, is beyond the scope of this paper, some preliminary observations can be made. 

While some non-evidence-based claims were incorrect regarding the statistics used, it can also be seen how qualifying language can be used to support the scientific accuracy of the claims. For instance, an evidence-based claim toward fertility used more circumspect language, carefully qualifying that “endometriosis *can* cause possible infertility” (our emphasis). In contrast, a non-evidence-based claim toward orthodox medicine used definitive and absolute language, stating that “physiotherapy, psychology, analgesics and surgery *do not* relieve much pain” (our emphasis). In the evidence-based example, it can be seen how the language used by the content creator carefully notes how infertility is not a symptom that all people with endometriosis experience, whereas the non-evidence-based claim paints with a broader brush, rendering multiple types of therapies as not able to relieve much pain. However, endometriosis presents diverse symptoms [5,6], and while satisfaction with certain treatment options is low in Australia [7], this does not mean that these treatment options are not effective for all the people who choose to use them. What is also seen in these non-evidence-based and mixed claims (Table 2) is what we argue may be a mixture of narrative experiences and evidence, given that social media is often used to expose the challenges of living with chronic conditions and to relay personal experiences [32]. This is further explored in the discussion, as is the evidence-based nature of the most salient claims and categories found within this study. 

## 4. Discussion

This study investigated the evidence-based nature of content about endometriosis posted on Instagram, using a unique approach to social media analysis that has yet to be applied to Instagram content. It was found that only four categories (cure, scientific article, symptoms, and fertility) comprised claims that were at least 50% or greater evidence-based. This aligns with previous research that found content on Facebook about endometriosis to be largely evidence-based [19]. More commonly, however, we identified that “evidence-based”, “non-evidence-based”, or “mixed evidence” claims varied between different content categories, with surgery, diagnosis, and orthodox treatments having the most non-evidence-based claims.

The “partly accurate” or “mixed” claims were often seen where narrative experiences, such as anecdotal stories about people’s experiences with their endometriosis, were combined with claims to scientific or medical evidence. In this sense, endometriosis-related information on Instagram undergoes and is sifted through multiple channels of communication and is at times narrativised via people’s individual experiences. Thus, despite the demonstrated engagement with scientific evidence, claims in a variety of categories often included misinterpretations, misunderstandings, or misapplications of that evidence, leading to the dominance of “mixed” claims. These misinterpretations or misunderstandings ranged from relatively minor errors to claims with major deviations from factual information with only small components of evidence-based content. This is an important finding, given that social media plays an important role in educating people about the disease, and that such platforms have the capacity to both reflect and exacerbate issues in caring for those with endometriosis [18,20].

An example of this “mixed” information was related to Zoladex, and was largely seen in the category of orthodox medicine. The primary mechanism of action of the drug is via inducing a hypoestrogenic state that results in menopause-like symptoms, carrying potential side effects such as reduced bone mineral density, and therefore generally restricted to short-term use [33]. Some claims related to Zoladex were evidence-based: “GnRH agonists have long term, sometimes irreversible effects on the body and should only be used as treatment to manage endo[metriosis] after diagnosis and with patient consent”, but these were vastly outnumbered by claims that referred to Zoladex as causing “chemical menopause”. What was somewhat surprising was that comments such as “Zoladex is forced on those with endometriosis” were often made specifically in relation to the Australian RANZCOG guidelines via the #changetheguidelines hashtag. However, the RANZCOG guidelines themselves state “As an *adjunct* to surgery for deep endometriosis… *consider* 3 months of gonadotropin-releasing hormone (GnRH) agonists before surgery” (authors’ emphasis). These reports of being “forced” to try Zoladex may be explained by a number of circumstances; these treatments may have been recommended prior to publication of the guideline or by a treating doctor who did not follow (or was not aware of) the advice within the guidelines. In this sense, claims pertaining to Zoladex may be a reflection of the personal experiences of the content creator, rather than what is said within the guidelines themselves. 

The categories of epidemiology and pathophysiology also largely comprised mixed claims. The changing nuances of scientific language may have been a contributing factor to this result, and consequently presents an ongoing challenge for those in the endometriosis community. For example, in defining endometriosis, claims often referred to endometriosis as a disease “where the tissue similar to the kind that normally lines the uterus (called the endometrium) grows outside of your uterus where it doesn’t belong” while other posts used phrasing such as “that occurs when tissue that lines the uterus grows outside it causing pain or infertility”. The description of “tissue that lines the uterus” versus “endometrial-like” or “tissue similar to the lining of the uterus” has been discussed heavily in the literature [34,35,36,37,38]. While there are some features that are similar between the tissues, i.e., the endometrium of an individual with endometriosis (eutopic, inside the uterus) versus the endometriosis lesion (ectopic, outside of the uterus), there are also obvious functional and histological differences [39]. This adds an additional layer of complexity for those trying to explain and communicate endometriosis online. Similarly, the language around epidemiology within the posts also varied, describing prevalence with phrases such as “1 in 10 people”—which, due to the word “people”, overestimates the prevalence. Posts also stated “endo[metriosis] affects 1 in 10 people with a gynaecological system” and “despite affecting 1 in 10 born with a uterus, living with endo[metriosis] is an incredibly lonely experience”. Others have also found this “1 in 10” statistic common in Instagram posts, writing that this is scientifically supported knowledge given “current available research that suggests endometriosis impacts at least 10% of people with a uterus at reproductive age” [15]. However, the accuracy of language when discussing prevalence is important, and using the term “people with a uterus” was considered to be less than ideal, as it is possible to have endometriosis after a hysterectomy [40] as endometriosis by definition is an extra-uterine disease. It is also important to recognise that not all people with endometriosis identify as women [41,42], and there are rare cases in which endometriosis is found in cis-men [43]. Therefore, the changing language around endometriosis, and who it impacts, may also lead to misunderstandings of the disease, for instance, the belief that it cannot impact people who do not have a uterus. 

The prominence of mixed claims may be due to the notion of “expert patients” and how people with endometriosis embody their illness and build patient communities. Embodied health movements often challenge both medical and scientific authority, using the body as a counter-authority to “challenge science in its epistemological processes and its institutional form” [44], as was evidenced in the #changetheguidelines campaign. The endometriosis community has been described as an embodied health movement, encouraging the formation of a collective identity that is built around shared experience [45]. Social media platforms such as Instagram are an important element of this community-building, providing an arena for continuous dialogue and shared perspectives for those living with the disease [46]. People with endometriosis may also become expert patients, meaning they may have the skills and capacity to manage their own illness and condition, including recognising, monitoring, and responding to symptoms [47]. As patients move beyond being passive participants in their healthcare and become patient experts, advocates, or both, they become “equipped, enabled, empowered, and engaged in their own medical care” [32]. Because certain graduates of obstetrics and gynaecology programs may not be trained to handle the more severe and complex cases of endometriosis [48], there may be significant disappointment and disillusionment if people with endometriosis do not feel that their doctor is suitably knowledgeable. However, as our findings show, this does not mean that an expert patient automatically “gains the expertise of a physician” in terms of the ability to understand, keep abreast of, and interpret biomedical information and research, unless they have specific training in this area [49]. Therefore, patients should be cautious about advising others on diagnosis and treatment, including surgery. 

Our study found that surgery was the category with the most non-evidence-based claims. A large portion of these claims were discussions of ablation versus excision surgery—with many of the claims being variations on “excision is the gold standard treatment”. This is a particularly contentious issue which stems from several factors. The practice of referring to excision as the “gold standard” in these posts is problematic, as this implies a scientific consensus on best practice, based around randomised controlled trials [50], which is currently limited [51,52]. The goal of endometriosis surgery is to remove the endometriotic lesions [38], and many endometriosis surgeons use excision, as this can provide both lesion removal and histological confirmation of pathology [53,54]. However, there may be circumstances in which the lesions may be more suitably treated with ablation—for instance, on the surface of the ovary—and in which excision may cause more harm [55,56]. Confounding the matter of a “gold standard” is that few studies compare the benefits of excision over ablation. Only two studies have compared these two techniques directly, in which similar pain reductions were reported across both groups, with only dyspareunia being more significantly improved for excision over ablation [57,58]. Unfortunately, even “complete excision” undertaken by an expert endometriosis surgeon may result in substantial recurrence rates over time, with almost a third of people (28%) requiring repeat surgery by 10 years after their initial surgery [59]. It is also unclear if recurrence of symptoms is necessarily always related to disease recurrence [59]. Moreover, similar recurrence rates for excision versus ablation of endometriomas have been reported [60]. Therefore, surgery outcomes and rates of recurrence may be more dependent on multiple other possibly interacting parameters such as the type, severity, and location of endometriosis, the classification as superficial or deep [60], the skill of the surgeon, and/or the biological nature of the disease. Therefore, while it is understandable for those with endometriosis to want access to the treatment with the best short- and long-term outcomes, many of the claims circulated on Instagram do not reflect the current scientific evidence. A related issue is that many posts mentioned the importance of seeing an “excision surgeon”. This is not the title of a specialty in Australia or New Zealand, where training to become a minimally invasive gynaecological surgeon (MIGS)—including the advanced laparoscopic surgery for endometriosis—is governed by the Australasian Gynaecological Endoscopy & Surgery Society (AGES). The American, British, Canadian, and European equivalents (American Association of Gynecologic Laparoscopists, British Society for Gynaecological Endoscopy, Canadian Society for the Advancement of Gynecologic Excellence, and European Society for Gynaecological Endoscopy, respectively) also do not include “excision surgeon” as a specialist title. Conversely, any gynaecologist may in theory adopt this title of “excision surgeon”, but there is no formal qualification associated with it. 

We speculate that this may be contributing to the mainstreaming of both the terms “excision surgeon” and “gold standard”, and, therefore, the use of this term by the endometriosis community. Gynaecologists in the United States and Europe frequently use the term “excision specialist” when marketing their services online, and this discourse has transcended borders, as seen through this analysis. It has also been suggested that this narrative is possibly being spread through popular advocacy groups, such as Nancy’s Nook [61]. Those with endometriosis are often displeased with the care from their physicians, or have difficulty gaining a diagnosis, and thus social media platforms provide a place of support and education [6,18,19]. People with endometriosis often feel dismissed by medical professionals, as pain and other symptoms may be taken less seriously, contributing to diagnostic delay [62]. Therefore, the idea of an “excision surgeon”—presented to the community by gynaecologists marketing their services—offers people with endometriosis an opportunity to reclaim their health and wellness. However, given the fact that “excision specialist” is not a specialist title in Australia or abroad, the pervasiveness of this title on Instagram may be potentially confusing (and harmful). People may be looking for this specific title when deciding on which surgeon to see, and become disheartened when they do not find one (leading to delay in diagnosis or treatment). This may also further confuse people on which medical professionals are educated and qualified to treat endometriosis. There does not seem to be a simple solution to combatting this kind of discourse, with surgeons reporting that they need to spend significant time fighting such “misinformation” [61] while trying to discuss the importance of considering medical options as well as surgical ones.

Furthermore, claims to “excision surgery” were also duplicated and repeated across the dataset in a variety of posts made by different users. Prior research has noted that the spread of health-related misinformation is exacerbated by social media, which forms information silos and creates an echo-chamber effect [63]. Where misinformation is pervasive online, users aggregate around this shared belief, causing such information to become more “viral”, or common [64]. This was seen consistently in this study, not only in the duplication of “excision as the gold standard” and references to “excision surgeon” but pertaining to other topics as well. The pervasiveness of duplicated information is an important finding, given that people who belong to, or engage with, groups and information on Instagram about endometriosis are being exposed to this echo-chamber dialogue, and therefore they may be more reluctant to accept information during a clinical encounter if it counteracts group thinking [65]. 

The lag that can occur between changes in the literature and their dissemination into the community may also be a barrier for evidence-based information, as seen in the category of diagnosis. A common claim was “endo[metriosis] can only officially be diagnosed through invasive and expensive laparoscopic surgery or biopsy”, and other similar claims that indicated people did not consider any other form of diagnosis to be accurate or even possible. One possible reason for this is that surgery was until very recently considered the only reliable method to rule in endometriosis. Changes in the less-invasive diagnostic process are relatively new, with the ESHRE guidelines on endometriosis diagnosis being the first to overtly state that non-invasive imaging tests are reliable to rule in endometriosis [13]. Conversely, the ESHRE guidelines also correctly state, based on current evidence, that endometriosis cannot be ruled out with imaging and there is a still a role for surgery as a diagnostic tool, but it should no longer be the first option for diagnosis. Other research on the uptake and awareness of non-invasive diagnostic tests reveals that knowledge and utilisation are far behind the true potential of imaging [5,66]. Our findings indicate that healthcare providers, physicians, and health communicators for endometriosis may need to consider the rapidly changing landscape of endometriosis, and the time it takes for patient communities to become informed on new medical practices. It is also likely that some of those posting on social media may have been incorrectly informed that they do not have endometriosis, on the basis of a “negative” scan, yet they may then have been diagnosed with endometriosis at a later surgery. This experience would then reinforce concerns about the usage of other diagnostic methods. Therefore, health professionals need to be mindful of the current limitations to less-invasive diagnosis and ensuring that patients are not incorrectly “ruled out” by a negative scan. 

Given the potential for misunderstandings and misinterpretations of biomedical research, there is a need for health organisations and professional medical associations to engage in social media conversations. This is essential for building trust, as well as being able to correct misinterpretations of medical information. Trust is central to effective health communication, with the need for health communicators to build and sustain trusting relationships over long periods of time [67,68]. As noted by Schulz and Nakamoto [49], the experience of illness is both frightening and frustrating, not only because of the symptoms but also because it entails a loss of control. For endometriosis communities, communicators need to be mindful that these groups are often experiencing pain, distress, fear, and uncertainty, and may have had negative medical encounters and/or misdiagnoses accompanied by delayed treatment [62] and their trust in expert medical voices may be low [69]. When such factors are combined with health misinformation, mistrust can be exacerbated by indicating that there is no scientific consensus or that the official medical sources of information are not credible [70]. Existing research has argued for a prioritisation of research that enhances surveillance, investigates psychological drivers, assesses real-world consequences, and develops effective responses, in order to enable health communicators to better understand and respond to health misinformation [70].

Social media platforms like Instagram offer both barriers and opportunities for communicating effectively with online endometriosis communities. Some studies have shown the effectiveness of peer groups and expert organisations addressing misinformation via correctional messaging without damaging their credibility with social media audiences [71,72,73]. This can depend on *who* is doing the correcting and *how* the information is delivered, and the group’s level of trust towards that expert voice [74]. The social media community environment for endometriosis is not a landscape where voices of expert authority can simply intervene with a solution to provide the correct answers and the best evidence-based information. Rather, such expert voices need to be attentive to the needs and values of online endometriosis communities, how they source and interpret medical information about the disease, how they understand and experience endometriosis, and their levels of trust in different organisations and experts. It is also important to note that high-quality or over-complex information on social media may be difficult for lay audiences to understand [6] and that there is a prevalence of false information which those with endometriosis are finding online [16,21]. As identified by this study, experts wanting to engage with the endometriosis community need to be aware that certain information silos relating to endometriosis are prevalent on Instagram, which may directly impact the clinician–patient interaction if information goes against group knowledge or group thinking [65]. Health communicators also need to fully grasp the social media eco-system in which endometriosis communities congregate, in order to more effectively engage in dialogue with them. It is well recognised by health literacy scholars that communities need to feel supported and empowered to act on their knowledge with the potential to collaborate with experts [75,76], considering endometriosis communities frequently use social media to communicate information and support each other [6,18]. 

As Holowka’s study noted, online support groups and Instagram offer important spaces for social and communal practices amongst those with endometriosis—along with meaning-making, storytelling, and advocacy [18]. Indeed, through their discourse on Instagram, people with endometriosis are influencing discussions about the illness, including challenging the simplistic ways that it has been addressed, which could help improve the time to diagnosis [18]. Importantly, shared experiences are highly valued by many living with endometriosis, and may be helpful to others needing to understand their own experiences of the disease [18]. This draws attention to some of the positive and productive reasons why people with endometriosis might be engaging on social media platforms to share information and experiences (which may not be evidence-based). These digital communities offer more than just a platform for education and as resources, but exist as an important tool to build and sustain community, and allow those to share their story. 

This study is not without its limitations, including the fact that it only focused on Instagram content. Videos were also excluded from the dataset, and while this is common in studies investigating Instagram content about endometriosis [15], research which is focused on Instagram videos, as well as other social media platforms such as TikTok, is an important place of future inquiry. Furthermore, due to the nuances of social media as a dialogic tool, some information was difficult to interpret. For instance, sarcasm in posts was difficult to decipher, as was the mix of scientific information with narrative experiences. Therefore, further research is needed to investigate the impact of non-evidence-based information dominating digital discourses about endometriosis on healthcare experiences, as well as qualitative communications research to better understand the information found online about endometriosis, and why, and how, this information is shared.

## 5. Conclusions

Social media platforms such as Instagram are increasingly being used to share information about endometriosis. Given that people are making healthcare decisions based on posts made on various forms of social media, health communicators, clinicians, and those wanting to engage with the endometriosis community such as support and advocacy groups need to be aware of the increased reliance on social media as a tool for education, support, and sharing information about the disease. They also need to be aware of the type of information circulating in these spaces. Our content analysis indicated that information about endometriosis on Instagram varies in accuracy from evidence-based to mixed evidence to non-evidence-based information across several categories. Certain categories were dominated by non-evidence-based information, in which incomplete or inaccurate claims were constantly repeated by various users, demonstrating the “echo-chamber” quality of social media [63], which may have real-world effects [65]. In the case of surgery, a common claim repeated was “excision is the gold standard”, which may reinforce an inflated sense of the effectiveness of “excision surgery” as a stand-alone modality in contrast to the best available data. Mixed evidence claims were also frequently observed, whereby parts of a claim were supported by scientific evidence but often slightly misinterpreted, or mixed with narrative experiences. This was often seen in the categories of pathophysiology and epidemiology, in which information is complex to understand for those without specific training, and therefore potentially easy to misinterpret. Therefore, we conclude that expert voices need to be both attentive to the needs and values of online endometriosis communities and also ensure that accurate and translatable information is provided. It is important too that a single piece of evidence may only be a small part of a bigger picture of the research about endometriosis, and when taken out of context, it is not necessarily completely “correct”. Given that people with endometriosis are making healthcare decisions that may be strongly influenced by these posts, health communicators, clinicians, and support and advocacy groups need to be aware of the current dialogues being spread on social media, while also ensuring that accurate information is vital. 

While this study, along with the other literature on social media and endometriosis, is an important step in understanding the evidence-based nature of content about endometriosis online, further research is needed. Given the fact that we found a considerable amount of mixed and non-evidence-based claims, future research prospectively determining the impact of social media on medical decision-making in those with endometriosis would provide further insight into the real-world consequences of potentially misleading content. Furthermore, the categories that were more evidence-based should also be investigated, especially from a strategic communications perspective, to better understand how some information is interpreted and understood “correctly”, whereas other information is not. 

## Figures and Tables

**Table 1:** Number of claims within Instagram posts of an educational-content nature, listed in order from highest to lowest percentage (%) of evidence-based claims.

Category	Total No. of Posts	Total No. of Claims Overall	Total No. Evidenced Based Claims	Total No. Non-Evidenced-Based Claims	Total No. Mixed Claims
**Cure**	38	45	31 (68.88%)	10 (22.22%)	4 (8.88%)
**Scientific Article**	10	11	7 (63.63%)	1 (9.09%)	3 (27.27%)
**Symptoms**	134	145	86 (59.31%)	19 (13.10%)	40 (27.5%)
**Fertility**	44	44	24 (54.54%)	8 (18.18%)	12 (27.27%)
**Other**	6	7	3 (42.85%)	2 (28.57%)	2 (28.57%)
**Diagnosis**	65	76	28 (36.84%)	21 (27.63%)	27 (35.52%)
**Orthodox Medicine**	38	50	17 (34%)	17 (34%)	16 (32%)
**Epidemiology**	68	75	22 (29.33%)	21 (28%)	32 (42.66%)
**Surgery**	50	58	11 (18.95%)	35 (60.34%)	12 (20.68%)
**Pathophysiology**	86	114	21 (18.42%)	10 (8.77%)	83 (72.80%)
**Alternative Medicine**	31	56	6 (10.71)	18 (32.14%)	32 (57.14%)

**Table 2:** Example of claims from Instagram posts about endometriosis.

Example Claim		
Evidence-Based	Non-Evidence-Based	Mixed
*Endo[metriosis] can cause possible infertility* (Fertility).	*Physiotherapy, psychology, analgesics and surgery do not relieve much pain* (Orthodox Medicine).	*If a patient has suspected endo[metriosis], they MUST be referred to an excision specialist* (Surgery).
*For some, it [endometriosis] can prove to be excruciating, disrupting their normal routines and forcing them to plan their days around the pain* (Symptoms).	*The pill does not ‘fix’ anything and does not supress bleeding for those with endo[metriosis]* (Orthodox Medicine.)	*It’s [endometriosis] a chronic health issue that affects one in ten women and it’s hardly ever talked about* (Epidemiology).
*It takes on average eight years to get diagnosed* (Diagnosis).	*20–25% of endo[metriosis] patients affected by infertility may be asymptomatic* (Fertility).	*Research has found that high levels of depression and anxiety can also exacerbate the symptoms of this condition. This is due to an increased pain perception which occurs with these psychological conditions* (Scientific Article).
*There is no cure for endometriosis* (Cure).	*We do not need research into alternative therapies, MRI studies are useless* (Alternative Medicine).	*If a patient has endo[metriosis] so severe it can be seen on a DIE scan, it should be removed to prevent frozen pelvis and organ dysfunction and a range of other potential comorbidities* (Pathophysiology).

## Data Availability

The data presented in this study are available on request from the corresponding author. The data are not publicly available due to the ethics protocol of this study.
